# Objective activity tracking in spine surgery: a prospective feasibility study with a low-cost consumer grade wearable accelerometer

**DOI:** 10.1038/s41598-020-61893-4

**Published:** 2020-03-18

**Authors:** Martin N. Stienen, Paymon G. Rezaii, Allen L. Ho, Anand Veeravagu, Corinna C. Zygourakis, Christy Tomkins-Lane, Jon Park, John K. Ratliff, Atman M. Desai

**Affiliations:** 1 Department of Neurosurgery, Stanford University Hospitals and Clinics, Stanford California, USA; 2 Department of Neurosurgery, University Hospital Zurich, Zurich, Switzerland; 3 Clinical Neuroscience Center, University of Zurich, Zurich, Switzerland; 4 Wearable Health Lab, Department of Physical Medicine and Rehabilitation, Stanford University Hospitals and Clinics, Stanford California, USA; 5 Department of Health and Physical Education, Mount Royal University, Calgary, Alberta Canada

**Keywords:** Physical examination, Rehabilitation, Outcomes research, Neuropathic pain, Skeletal muscle

## Abstract

Patient-reported outcome measures (PROMs) are commonly used to estimate disability of patients with spinal degenerative disease. Emerging technological advances present an opportunity to provide objective measurements of activity. In a prospective, observational study we utilized a low-cost consumer grade wearable accelerometer (LCA) to determine patient activity (steps per day) preoperatively (baseline) and up to one year (Y1) after cervical and lumbar spine surgery. We studied 30 patients (46.7% male; mean age 57 years; 70% Caucasian) with a baseline activity level of 5624 steps per day. The activity level decreased by 71% in the 1^st^ postoperative week (p < 0.001) and remained 37% lower in the 2^nd^ (p < 0.001) and 23% lower in the 4^th^ week (p = 0.015). At no time point until Y1 did patients increase their activity level, compared to baseline. Activity was greater in patients with cervical, as compared to patients with lumbar spine disease. Age, sex, ethnic group, anesthesia risk score and fusion were variables associated with activity. There was no correlation between activity and PROMs, but a strong correlation with depression. Determining activity using LCAs provides real-time and longitudinal information about patient mobility and return of function. Recovery took place over the first eight postoperative weeks, with subtle improvement afterwards.

## Introduction

Patients with degenerative spine disease typically suffer from pain and functional disability, which negatively impact their activity levels and health-related quality of life (HRQoL). For a comprehensive patient evaluation, a variety of patient-reported outcome measures (PROMs) can be applied. These multi-dimensional questionnaires are considered the gold-standard of self-assessment^1,2^. However, these self-reported outcome measures are, at best, an approximation of pain and disability before and after surgery^3^.

More recently, a variety of functional tests have increasingly been applied to quantify objective functional impairment (OFI)^4–7^. In a systematic review of the literature, accelerometer and physical activity analyses using wearable devices ranked among the top five most frequently applied methods for objective outcome measurement in spine care^4^. To date, the activity levels of about 340 patients with lumbar degenerative diseases of the spine have been reported in nine research articles, which included patients with low back pain (LBP), lumbar spinal stenosis (LSS) and lumbar disc herniation (LDH)^8–16^.

Despite its great potential to contribute to the comprehensive patient evaluation, the current literature on activity tracking in spine patients remains limited. A recent systematic review confirmed that additional studies are needed to determine the strengths and weaknesses of using wearable devices in spine surgery^17^. To our knowledge, no prior studies have evaluated the use of wearable activity monitors to track postoperative recovery in patients with diseases of the cervical spine. Furthermore, prior studies involving patients with lumbar disorders have had small sample sizes and were restricted to short follow-up periods^4,17^. This study aimed at determining the feasibility of a low-cost consumer grade accelerometer (LCA) for objective assessment of pre- and postoperative step activity.

## Methods

In a prospective feasibility study, we utilized LCAs to provide longitudinal activity-based outcome measurements for patients undergoing elective spine surgery. The study was approved by the institutional review board at Stanford University.

### LCA technology

LCAs utilize three-dimensional accelerometers to sense user movement and can continuously measure home and community activity levels. For our study, the Mi Band (Xiaomi, Mountain View, CA (USA)) was utilized. While no device-specific data is available, a body of literature supports the notion that wearable LCAs are generally reliable and are valid indicators of overall physical activity in adults^4^.

At the time of study inclusion, patients were educated on LCA use by a study physician, including how to properly wear, maintain the device, and sync data to their mobile phones with Bluetooth technology. Patients were encouraged to wear the device as much as possible, though voluntary removal of the device for periods of time did not lead to exclusion from the study. The device was interrogated by study staff for preoperative data extraction and removed on the day of surgery. The device was reapplied immediately post-operatively. Data was extracted again on the day of discharge as well as at each follow-up visit for up to one year post-operatively.

### Study Population

Over a 9-month period, we screened adult patients (≥18 years) presenting at our department’s outpatient spine clinic for the first evaluation of a degenerative condition of the subaxial cervical, thoracic or lumbar spine. We offered participation to those owning an iOS or Android smartphone (for compatibility with the Mi Fit app), scheduled for an elective surgical procedure under general anesthesia in more than one week.

### Data collection

Activity tracking was conducted longitudinally. PROMs were assessed in the 30-day period before surgery (baseline), as well as at clinic visits at 3 months (M3) and 1 year (Y1) postoperatively. For this, we used our department’s integrated electronic medical record database that collects PROM data^18^, including a screening for depression using the Patient Health Questionnaire (PHQ-)2 for all patients, the Oswestry Disability Index (ODI) for patients with lumbar spine conditions and the Neck Disability Index (NDI) for patients with cervical spine disease.

The ODI and NDI are the most commonly used PROMs for patients with degenerative diseases of the spine, as they include many relevant aspects of life (pain, personal care, common activities) and their reliability and validity has repeatedly been demonstrated^19–21^. The PHQ-2 is a brief multipurpose instrument for screening, diagnosing, monitoring and measuring depression in patients and ranges from 0–6 points, with depression likely for scores ≥ 3^22^.

### Statistical analysis

Our dependent variable of interest was the mean number of steps per day, determined at baseline, as well as 1, 2, 4, 8, 12 (M3), 26 and 52 weeks (Y1) postoperatively. Descriptive analyses were used to report activity data (daily steps; mean and standard deviation (SD)), as well as feasibility and safety.

Independent variables were patient age (in years), sex, body mass index (BMI; obesity defined according to the WHO as BMI ≥ 30 kg/m^2^), ethnic background, smoking status (stratified into active/former smokers vs. never smokers), diagnosis, type of procedure (decompression vs. decompression/fusion), number of operated levels (single, two-level or multiple levels), anesthesia risk (American Society of Anesthesiologists (ASA) risk scale; stratified into low (ASA 1 or 2) and high (ASA 3)), as well as disability measures (ODI & NDI) at baseline and follow-up (see Table 1). To evaluate for depressive comorbidity, PHQ-2 scores ≥ 3 were used as recommended cut-offs^22^. The independent variables’ association with the activity levels at baseline and/or follow-up was tested using linear regression, student’s t-tests and analysis of covariance (ANOVA) models. As we found a marked difference between the activity levels in patients with cervical and lumbar spine disease, we adjusted for this in subsequent analyses using multivariable linear regression or multivariable analysis of covariance (MANOVA) models. Pearson correlation coefficients were calculated between PROMs and activity levels. Analyses were conducted using Stata v14.2 (College Station, Texas (USA)).

**Table 1:** Basic demographic information.

Variable	Result
Age in years	57.1 (14.9)
**Sex**	
Female	15 (50.0%)
Male	15 (50.0%)
**Ethnicity**	
Caucasian	21 (70.0%)
Hispanic	4 (13.3%)
Black	2 (6.7%)
Asian	2 (6.7%)
Indian	1 (3.3%)
BMI in kg/m^2^	28.8 (4.9)
**Smoking status**	
Active smoker	1 (3.3%)
Former smoker	10 (33.3%)
Never smoker	19 (63.3%)
Depressive comorbidity *	
No	18 (60.0%)
Yes	11 (36.7%)
Unknown	1 (3.3%)
**Diagnosis**	
Lumbar spondylolisthesis	7 (23.3%)
LSS	8 (26.7%)
LDH	3 (10.0%)
CDH with radicular pain (soft)	4 (13.3%)
Cervical foraminal stenosis with radicular pain (hard)	3 (10.0%)
Cervical myelopathy for any reason	5 (16.7%)
**Surgical procedure**	
Lumbar discectomy/decompression w/o instrumentation/fusion	9 (30.0%)
Lumbar decompression w/ instrumentation/fusion	9 (30.0%)
Anterior cervical discectomy (and fusion) **	9 (30.0%)
Posterior cervical decompression (and fusion)	3 (10.0%)
**ASA risk scale**	
1	2 (6.7%)
2	18 (60.0%)
3	10 (33.3%)
**Levels treated**	
1	12 (40.0%)
2	13 (43.3%)
≥3	5 (16.7%)
Preoperative steps per day	5624 (2776)
**Total**	**n = 30 (100%)**

Results are presented as mean (SD) or count (%). ASA = American Society of Anesthesiologists; BMI = body mass index; CDH = cervical disc herniation; LDH = lumbar disc herniation; LSS = lumbar spinal stenosis.

*As determined by PHQ-2 ≥ 3 points.

**The group included two patients with motion preserving total disc arthroplasty.

According to the predefined protocol, we aimed to enroll at least 20 patients in this study. Findings with *p* ≤ 0.05 were considered statistically significant.

### Ethical approval and informed consent

The study was approved by the institutional review board (IRB) at Stanford University and conducted in accordance with the ethical standards of the institutional and/or national research committee and with the 1964 Helsinki declaration and its later amendments or comparable ethical standards. All patients signed written informed consent prior to inclusion.

## Results

### Feasibility

In the 9-month period, a total of 3742 outpatient spine clinic visits were conducted, of which 1614 were new patient visits. 607 (37.6%) eligible patients were scheduled for elective spine surgery. Of those, 48 patients were asked to participate (7.9%) and 42 were included. Seven were excluded for non-compliance with the study protocol and in 5 the planned surgery was cancelled. Therefore, 87.5% of patients (42/48) asked to participate in this study consented, and 81.1% of patients (30/37) who consented and underwent surgery provided objective outcome data.

### Patient characteristics

Patient demographics are described in Table 1 (n = 30 patients; 46.7% male; mean age: 57.1 ± 14.9 years; 70% Caucasian). The mean baseline activity level was 5624 steps per day (SD 2776; median 5063). Objective outcome data was available for the entire cohort at baseline and at postoperative weeks 2 and 4. 29/30 patients provided outcome data at postoperative week 1, 26/30 at postoperative weeks 8, 21/30 at postoperative weeks 12 (M3) and 26, and 11/30 patients until the Y1 time point. There were no adverse events related to the device.

### Activity tracking

**Figure 1:**
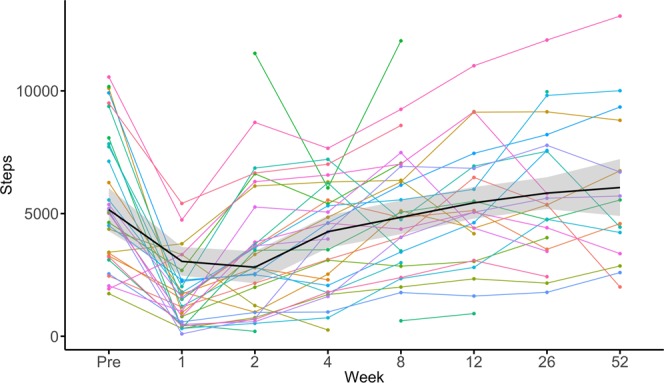
The line graph illustrates the absolute change in the daily steps for the total cohort of n = 30 patients with spine diseases. The x-axis indicates the time from preoperative (Pre) to 52 weeks postoperative. The y-axis indicates the number of steps per day. Individual patient data is illustrated as colored line. The mean of the total cohort is marked as black line with grey 95% confidence interval.

**Figure 2:**
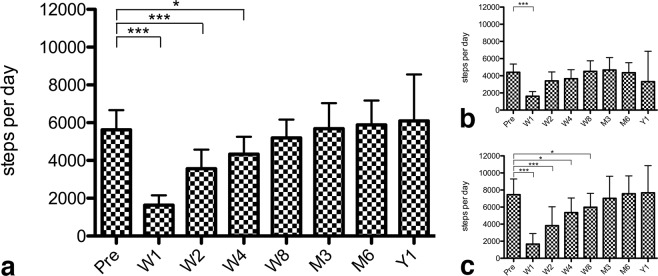
Bar graphs with whiskers (95% confidence interval), illustrating the absolute change in the daily steps of the total cohort (**a**), n = 18 patients with lumbar spine diseases **(b)** or n = 12 patients with cervical spine disease (**c**). The x-axis indicates the time from preoperative (Pre) to 52 weeks postoperative. The y-axis indicates the mean number of steps. The levels of significance are indicated as *(*p* ≤ 0.05), **(*p* ≤ 0.005) or ***(*p* ≤ 0.001).

Figure 1 illustrates the change in the number of daily steps for the whole cohort. Compared to baseline, the activity level decreased by 71% to an average of 1635 steps per day (SD 1376; median 1219) in the 1^st^ postoperative week (p < 0.001). It remained 37% lower in the 2^nd^ (mean 3569, SD 2694; p < 0.001; median 3226) and 23% lower in the 4^th^ postoperative week (mean 4335, SD 2469; p = 0.015; median 4487), but the difference lost statistical significance from the postoperative week 8 onwards until Y1 (Fig. 2a; all p > 0.05).

### Cervical vs. lumbar disease

The mean daily number of steps of 18 patients with lumbar spine disease was 4403 (SD 1936; median 4055) at baseline. This activity decreased by 63% to 1615 (SD 1078; p < 0.001; median 1362) in the first postoperative week. From postoperative week 2 onwards throughout the remaining 1-year follow-up, the number of daily steps was within the range of the baseline assessment (all p > 0.05; Fig. 2b).

The mean daily number of steps of 12 patients with cervical spine disease was 7455 (SD 2903; median 7866) at baseline. This number decreased by 78% to 1667 (SD 1824; p < 0.001; median 953) in the first postoperative week and remained 49% lower during postoperative week 2 (mean 3820; SD 3491; p < 0.001; median 3226), 28% lower during postoperative week 4 (mean 5359; SD 2692; p = 0.015; median 5372) and 20% lower during postoperative week 8 with borderline significance (mean 5983; SD 2559; p = 0.050; median 6207). From M3 onwards throughout the remaining 1-year follow-up, the number of daily steps was within the range of the baseline assessment (all p > 0.05; Fig. 2c).

The mean daily number of steps was significantly greater for the cohort of patients with cervical, as compared to those with lumbar spine disorders at baseline (p = 0.002), as well as on postoperative weeks 26 (p = 0.006) and 52 (Y1; p = 0.05). Activity was comparable at the remaining time points (all p > 0.05).

### Factors associated with baseline activity level

Patients with low ASA grade were significantly more active than patients with high ASA grade (6263 ± 2713 (SD) vs. 4345 ± 2562 (SD); p = 0.033). None of the other factors, such as age (p = 0.107), sex (p = 0.104), ethnic group (p = 0.107), obesity (p = 0.140) or smoking status (p = 0.729) were associated with baseline activity levels.

### Factors associated with early postoperative mobility

In the first postoperative week, there was a tendency for 32 steps less per day for every year increase in patient age (Coef. −32, 95% CI −66 to 2; p = 0.067). The mean activity differed between ethnic groups (p = 0.039), with highest to lowest activity levels observed in patients of Indian, Black or African-American, Hispanic, Caucasian and Asian ethnicity. There was no significant difference in the number of steps per sex, obesity, smoking status, anesthesia risk or the number of operated levels (all p > 0.05). However, patients who underwent a spinal fusion procedure had a 48% lower activity level (1213 ± 1219 (SD) vs. 2325 ± 1390 (SD); p = 0.032) at the first postoperative week.

In the second postoperative week, every year increase in patient age was associated with lower patient activity by 75 steps (Coef. −75, 95% CI −138 to −11, p = 0.022). No other variable was found to be significantly associated with the number of steps (all p > 0.05).

At the postoperative week 4, 8 and at M3 time points, none of the variables were significantly associated with activity level.

### Factors associated with long-term postoperative activity

At postoperative week 26, none of the variables of interest were significantly associated with patient activity. At the Y1 time point, there was a tendency for 158 steps less per day for every year increase in patient age (Coef. −158, 95% CI −328 to 12; p = 0.065), and female patients were more active than their male counterparts (8618 ± 2823 (SD) vs. 3058 ± 1575 (SD); p = 0.016). No other variables of interest were significantly associated with patient activity at Y1.

### Convergent validity with PROMs

As shown in Table 2 and Figs. 1 and 2a–c, as ODI/NDI values decreased at M3 and Y1, the activity levels of patients increased. There was no significant association between the activity level and the ODI at baseline or Y1 in patients with lumbar spine disease (all p > 0.05). There was a tendency for moderate negative correlation between the patients’ activity level and the ODI at M3 (r = −0.569, p = 0.054). There was no significant association between the activity level and the NDI at baseline, M3 or Y1 in patients with cervical spine disease (all p > 0.05).

**Table 2:** Subjective PROM and objective activity results at baseline (preoperative), as well as at postoperative week 12 (M3) and 52 (Y1).

Variable	Preoperative	M3	Y1	p-value*
Cervical				
Steps per day, mean (SD)	7455 (2903)	7020 (3376)	7678 (3432)	^#^*p* = 0.755 ^$^*p* = 0.119
NDI, mean (SD)	37.8 (14.9)	27.9 (11.7)	23.5 (16.3)	^#^*p* = 0.071 ^$^*p* = 0.036
PHQ-2, mean (SD)	2.3 (2.1)	1.5 (1.9)	0.8 (1.6)	^#^*p* = 0.555 ^$^*p* = 0.129
Steps-ODI, Pearson correlation	r = 0.063 p = 0.803	r = −0.046 p = 0.907	r = −0.083 p = 0.876	
Steps-PHQ-2, Pearson correlation	r = −0.014 p = 0.957	r = −0.739 p = 0.036	r = −0.331 p = 0.785	
**Lumbar**				
Steps per day, mean (SD)	4403 (1936)	4679 (2265)	3312 (2228)	^#^*p* = 0.535 ^$^*p* = 0.179
ODI, mean (SD)	45.1 (16.6)	27.1 (17.6)	25.5 (17.3)	^#^*p* = 0.004 ^$^*p* = 0.005
PHQ-2, mean (SD)	1.9 (2.3)	0.3 (0.8)	0.7 (0.8)	^#^*p* = 0.396 ^$^*p* = 0.083
Steps-ODI, Pearson correlation	r = −0.164 p = 0.612	r = −0.569 p = 0.054	r = −0.075 p = 0.926	
Steps-PHQ-2, Pearson correlation	r = −0.525 p = 0.080	n/a**	r = −0.762 p = 0.238	
**Total cohort**				
Steps-PHQ-2, Pearson	r = −0.274 p = 0.143	r = −0.739 p = 0.003	r = −0.474 p = 0.283	

The Pearson correlation coefficients (r) are illustrated to describe the relationship between steps per day and the ODI for patients with lumbar spine disease, and steps per day and NDI for patients with cervical spine disease at all three time points.

*^#^for comparison of preoperative to M3 and ^$^ for comparison of preoperative to Y1.

**PHQ-2 was 0 points for all patients with available step data, rendering analysis impossible.

With regards to PHQ-2, there was a strong negative correlation between the patients’ activity level and the PHQ-2 score at M3 (r = −0.739, p = 0.003) for the total sample. There was no significant correlation between the activity levels at baseline or Y1 and the PHQ2 (all p > 0.05; see Table 2 for details).

### Case vignettes

**Figure 3:**
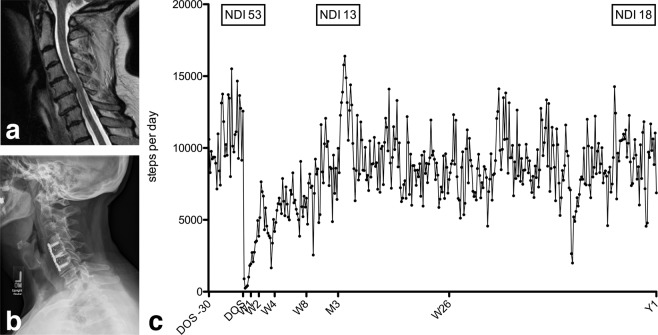
Case vignette of a 69-year old female (BMI 36.2 kg/m^2^; ASA 3), who underwent uneventful C4-6 ACDF for cervical spondylitic myelopathy with pain, numbness/tingling and progressive dexterity issues (NDI 53). (**a**) The preoperative sagittal T2-weighted MRI gives evidence of increased signal intensity at the C4/5 level, consistent with myelopathy, and cervical stenosis at the C5/6 level. (**b**) Lateral x-rays at Y1 demonstrate a regular postoperative situation after C4-6 ACDF. (**c**) Individual activity data is illustrated over time, from 30 days before the day of surgery (DOS), over postoperative weeks (W) 1, 2, 4, 8, 12 (M3), 26 and 52 (Y1). At her M3 follow-up visits, she states a nearly 100% improvement of her preoperative symptoms (NDI 13). At her Y1 follow-up visit, she was still significantly better compared to baseline, but she reported a fall from a ladder about 6 months postoperative with minor neck and shoulder pain ever since (NDI 18). The objective activity tracking illustrates remarkable recovery over the first 12 postoperative weeks, with stagnation of further process afterwards.

**Figure 4:**
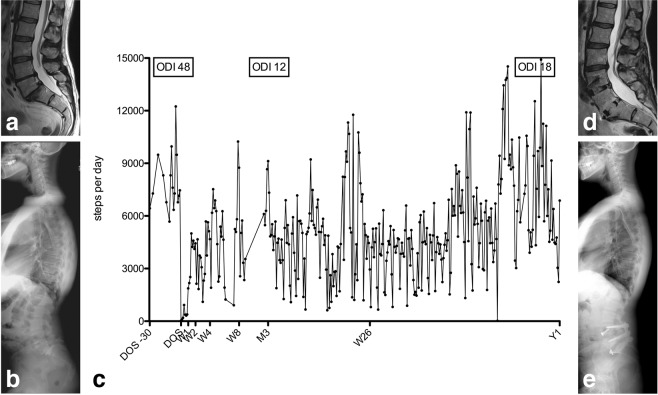
Case vignette of a 48-year old female former gymnast (BMI 27.6 kg/m^2^; ASA 2), who underwent uneventful L4-S1 single-stage anterior lumbar interbody fusion and posterior spinal fusion for intractable low back pain and bilateral lower extremity pain (left > right) due to degenerative disc disease at L4-S1 with grade-1 isthmic spondylolisthesis at L5-S1 (ODI 48). (**a**) Preoperative sagittal T2-weighted MRI. (**b**) Preoperative long-cassette x-ray show a high pelvic incidence of 70° and a lumbar lordosis (LL) of 62° with preserved sagittal vertical alignment (SVA) of +3.5 cm. (**c**) Individual activity data is illustrated over time, from 30 days before the day of surgery (DOS), over postoperative weeks (W) 1, 2, 4, 8, 12 (M3), 26 and 52 (Y1). At her M3 follow-up visit the patient reports being 95% better compared to preoperative (ODI 12) and her standing x-rays are unremarkable. At her Y1 follow-up visit, she reports new onset of mild low back pain and some left-sided leg pain, translating into lesser activity and mild increase in the ODI (18). (**d**) The MRI at Y1 shows a solid fusion at the L4-S1 levels with adjacent segment disease, mild disc protrusion and facet disease at L3-4. The patient improved after additional epidural steroid injection at the L3-4 level and so far no additional surgical treatment was required. (**e**) Post-operative long-cassette x-ray show a LL of 72°, SVA of +1 cm.

**Figure 5:**
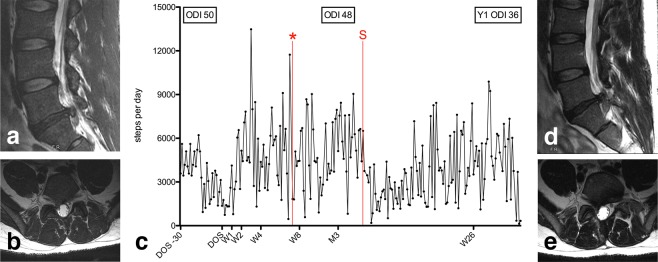
A 33-year old male patient (BMI 26.2 kg/m^2^; ASA 2) underwent an uneventful right L5/S1 microdiscectomy for right S1-lumboradicular pain resulting from LDH and non-responsive to conservative management (ODI 50). (**a**,**b**) Preoperative sagittal (**a**) and axial (**b**) T2-weighted MRI. (**c**) Individual activity data is illustrated over time, from 30 days before the day of surgery (DOS), over postoperative weeks (W) 1, 2, 4, 8, 12 (M3) and 26. Initially, he experienced complete resolution of his symptoms. After a decrease in the activity level at the 1^st^ postoperative week, he was able to regain (2^nd^ week) and almost double his baseline activity at the 4^th^ week. He then carried a 5-gallon (40-50lbs) jug of paint about seven weeks postoperative and noted new onset of low back and right-sided leg pain with a new plantarflexion weakness (*). A repeat MRI was consistent with a re-herniated right paramedian L5/S1 LDH (**d**) sagittal view; (**e**) axial view). The patient underwent repeat surgery (S) about 14 weeks after the initial surgery. The M3 ODI was 48 and the Y1 ODI 36. The patient decided against further objective step count measurements for this study beyond 217 days postoperative.

Illustrative case vignettes highlight the value of LCA-based activity tracking in patients with cervical spine disease (Fig. 3a–d), lumbar spine disease (Fig. 4a–e) and in a patient with a postoperative complication (Fig. 5a–e).

## Discussion

Increased physical activity can lead to better physical and mental outcomes^23–26^, and level of physical activity can serve as a useful measure of overall health^27^. Postoperative mobility has long been touted as a reliable indicator of surgical recovery and good post-operative prognosis^2,3,6,7,14,28–32^. Most mobility data, especially in the perioperative setting, take the form of subjective self-reported questionnaires that suffer from recall bias, conformation to socially desirable responses, and are heavily influenced by mood, depression, anxiety, cognition, and disability^3,33,34^. Clinical assessments are static, discrete evaluations that can provide some measure of functional ability only at a single time-point. There is a profound lack of data reporting mobility in the perioperative period^4,17,32^.

In a prospective observational setting, we evaluated the activity levels of a sample of 30 patients before and up to 1 year after elective spine surgery. Objective activity monitoring was safe and well-tolerated by patients. Patients with lumbar spine disease showed generally lower activity levels, compared to patients with cervical spine disease. Activity levels significantly decreased after surgery, and recovery to baseline activity levels took place over the course of several weeks in both patients with cervical and lumbar spine disease. Patients regained their peak activity around 12 weeks postoperatively (M3); additional meaningful improvement after that time was unlikely and patients generally did not improve beyond their preoperative baseline levels. Even though the step count generally increased with progressive recovery documented by the PROMs, the direct statistical correlations between subjective and objective outcome measures were mostly weak and insignificant.

### Feasibility and safety

Most patients (87.5%) who were approached about the study agreed to participate and the dropout rate of enrolled patients was fairly low (18.9%). Once included, no additional effort was needed to motivate patients continue wear the LCA, but we reminded patients to upload and send data at predefined intervals. The feasibility of using a LCA is supported by the high acceptance rate and affordability, making its distribution and the determination of solid objective activity results possible in a broader population. In the U.S., the Centers for Medicare and Medicaid Services (CMS) began reimbursing for remote patient monitoring in 2018 via the CPT code 99091, which is likely going to support the increasing use of objective outcome measures further.

There were no complications or (severe) adverse events recorded that could potentially be associated with the LCA use. The one patient who required repeat surgery for a re-herniated disc was involved in non-recommended high physical activity, as described in the case vignette (Fig. 5).

### Accuracy, reliability & responsiveness

It was not the aim of this study to investigate or report on reliability measures of the LCA device. Reports from users, who checked the device against other step counters generally testify its accuracy, and research with similar devices found accuracy rates of around 98%^10^. However, more research is required to determine this and other devices’ accuracy and (test-retest) reliability measures^17^.

We found the daily step count to be a very responsive marker of overall physical activity, reflecting the reduced activity levels in the first 8–12 weeks after surgery well. Changes in activity were subtle after this time period, which likely mirrors the more gradual further improvement and rehabilitation often described for PROMs in a similar manner^29,35^. Our findings are in line with a prior study that found improved PROMs but no higher physical activity than baseline, six months after successful surgery for LSS^36^. The LCAs appeared suitable to detect worsening or low-level activity in patients with obvious unfavorable clinical courses (Fig. 5c). Understanding of responsiveness remains limited as most studies on wearable activity tracking devices only assessed patients at one time point pre- or postoperatively^4,17^, or included other longitudinal measures but did not analyze daily steps^29^. This report adds new long-term postoperative outcome data to the current understanding in this emerging field of research. Our current and some previous findings on objective activity tracking indicate that spine surgery increases patients’ capacity and capability (as measured by PROMs), but in order to change lifestyle and increase patient activity beyond the preoperative level additional rehabilitative interventions are needed^36^.

### Association between LCA activity and PROMs

Cross-validating activity data with PROMs in lumbar spine patients (ODI) and cervical spine patients (NDI) revealed weak and non-significant correlation coefficients (Table 2). The current findings are in line with the results of prior studies suggesting weak to moderate correlations between subjective and objective outcome measures, if at all^4–6,10,29,31,37,38^. In their accelerometer study on n = 28 patients undergoing lumbar spine surgery, Mobbs *et al*. did not find a significant correlation between improvements in subjective clinical outcome and changes in physical activity measurements at follow-up^10^. Still, the increase in activity after surgery paralleling the recovery (Figs. 3c, 4c), or lack thereof (Fig. 5c) suggest that steps per day is a valid surrogate marker of outcome. Also, cervical spine patients started from and regained a higher activity level, and patients who underwent a fusion procedure were only half as active as those undergoing minimally-invasive decompression – both findings reflecting our daily clinical experience and suggesting that the objective outcome measurement is valid. They once more underline the fact that objective outcome measurement may not replace the subjective patient evaluation by PROMs, but rather contribute a new dimension to the comprehensive patient evaluation^5,6,38^.

It is likely that the small sample size, large inter-individual variability in activity, the heterogeneous patient sample, and the patient-specific time course of postoperative recovery made it difficult to formally ascertain the cross-validity of activity data with PROMs. Further studies employing LCAs in larger and more homogenous patient cohorts are needed to study this relationship in more detail. The fact that both patients after lumbar and cervical spine surgery showed a several week-long decrease in activity may indicate that the disability & mobility restriction results less from incisional pain but possibly from general postoperative fatigue or factors not assessed in this study.

It should also be noted that we commonly recommend patients to “take it easy” during the initial 6–8 postoperative weeks, and the reduced activity could be a sign of patient compliance rather than spine-related mobility restriction. After the first 6–8 weeks, all patients received physiotherapy (PT) prescriptions for isometric core muscle strengthening, followed by increasing range of motion and activity. The exact time of starting with PT was not recorded for the scope of this research and therefore its effect on activity could not be measured.

### Assessment in patients with depression

The mental health condition of patients with degenerative spine disease is known to bias the subjective, PROM-based outcome assessment^39–41^. One of the hopes of objective outcome measures is to provide an accurate determination of functional outcome, irrespective of the mental health condition^42^. In our patient population, depression was associated with reduced physical activity levels at M3 (r = −0.739, p = 0.003). Our findings are consistent with the literature. Ludwig *et al*. used the PHQ-9 to evaluate 1742 adult patients with cardiovascular disease and showed the daily step count of depressed patients differed significantly (p < 0.001) with moderate to severe depressive symptom patients walking 13.3% (95% CI 18.8%–7.9%) and 15.6% (95% CI 23.7%–6.5%) less^23^.

### Future implications

Recent advances have produced smaller, cheaper, lighter, and smarter devices that have been marketed directly to consumers and have gained widespread popularity^4,43^. Accelerometers are increasingly integrated into smartphones, smartwatches, and other wearable digital electronics. The objective determination of functional outcome is preferred over questionnaire-based evaluations by most patients with lumbar spine disease^44^, and tools for the unobtrusive longitudinal assessment of functional outcome have the potential to lower the missing data burden inherent to PROM-based research: an analysis of 13 large prospective spine registries found loss of follow-up rates to range between 21–78%^45^.

Incentive-based interventions made possible by immediate feedback, Internet or social media have been shown to effectively modify health behavior^15,46,47^. This is of particular interest for post-operative patients, as improvement in activity levels beyond the preoperative baseline was infrequent (Figs. 1 and 2a–c). Even though disability and pain was improved after surgery, our patients did not maximize the benefits of the intervention by walking more steps. This finding points towards the potential of rehabilitation strategies. Further potential lies in the early detection of complications and/or unfavorable treatment outcomes in patients with decline in activity or lack of postoperative improvement^48^.

It must be emphasized that the use of LCAs in spine health is likely still in its relative infancy. While PROMs remain the gold standard of outcome assessment, other objective outcome measures such as the Timed-Up and Go (TUG) test^5,37,38,42,49,50^, the 6-minute walking test (6WT)^7,29^, the 5-Repetitions Sit-To-Stand (5R-STS) test^6^ or the Self-Paced Walking Test (SPWT)^31,51^ are better studied and validated^4,17,32^. A wide range of activity tracking devices are available on the market enabling objective activity tracking as an experimental, additional outcome marker for now. Our present analysis suggests that daily steps are a sensitive, but nonspecific surrogate marker of spine-related mobility restriction.

Future randomized trails might benefit from implementing both subjective PROM-based and objective outcome measures in order to estimate patient outcome as accurately as possible and herewith support therapeutic decision-making.

### Strengths & limitations

This study adds new data to the existing literature on objective activity and outcome analysis. Despite our small sample size, we exceeded most previous similar studies with regards to included patients and length of follow-up^4,10,15,17,29,32,51,52^. In addition to the objective activity tracking, several well-validated PROMs were applied to assess outcome on subjective scales. Finally, this is the first study to include cervical spine patients and to compare activity levels of patients with degenerative lumbar versus cervical spine diseases.

Certainly, a weakness of this study is the small percentage of potentially eligible patients that were finally asked to participate in this study. The single most important reason behind non-inclusion was lack of dedicated study personnel; incompatibility issues between the LCA and smartphone device were infrequent. A further implication of the sample size and heterogeneity is that we refrain from going into more detail regarding the observed relationship of patient characteristics (e.g., ASA score, obesity, etc.) and objective mobility data. As with many other studies^35,45^, also this study was impacted by loss of follow-up and inherent missing data in the range of 0–30% at short and 3–63% at long-term follow-up. The inclusion of a heterogeneous patient sample allowed us to gain insights into recovery for a wide range of disease types. Further research should concentrate on characterizing short- and long-term recovery for more well-defined populations. Lastly, we utilized simplified summary data for the main analyses; a more detailed review of day-to-day activity data as illustrated in Figs. 3c, 4c and 5c might identify additional important factors impacting spine-related mobility.

## Conclusion

This prospective observational study provides novel insight into the objective activity levels of patients before and after cervical and lumbar spine surgery. Activity tracking provided evidence for progressive recovery over the first 8–12 postoperative weeks, but improvement beyond the preoperative activity level was uncommon up to the Y1 follow-up. The correlation between daily steps and subjective outcome measures was weak, indicating that activity tracking should not replace PROMs but rather add another dimension to the comprehensive outcome assessment of patients undergoing spine surgery.

## Data Availability

All data are available from the corresponding author upon reasonable request.
